# A 2-month field cohort study of SARS-CoV-2 in saliva of BNT162b2 vaccinated nursing home workers

**DOI:** 10.1038/s43856-021-00067-3

**Published:** 2022-01-10

**Authors:** Claude Saegerman, Anh Nguyet Diep, Véronique Renault, Anne-Françoise Donneau, Lambert Stamatakis, Wouter Coppieters, Fabienne Michel, Christophe Breuer, Margaux Dandoy, Olivier Ek, Claire Gourzones, Joey Schyns, Emeline Goffin, Frédéric Minner, Keith Durkin, Maria Artesi, Vincent Bours, Fabrice Bureau, Laurent Gillet

**Affiliations:** 1 Fundamental and Applied Research for Animal and Health (FARAH) Center, Liège University, Liège, Belgium; 2 Risk Assessment Group COVID-19, Liège University, Liège, Belgium; 3 Biostatistics Unit, Liège University, Liège, Belgium; 4 General Delegation COVID-19, Government of the Walloon Region, Namur, Belgium; 5 Unit of Animal Genomics, GIGA Institute, Liège University, Liège, Belgium; 6 Collection and Analysis of Data and Information of Strategic Utility (RADIUS), Liège University, Liège, Belgium; 7 Liège University, Liège, Belgium; 8 Covid-19 Platform of Liège University, Liège, Belgium; 9 Laboratory of Human Genetics, GIGA Research Institute, Liège, Belgium; 10 Laboratory of Cellular and Molecular Immunology, GIGA Institute, Liège University, Liège, Belgium; 11 Laboratory of Immunology-Vaccinology, Liège University, Liège, Belgium

**Keywords:** Epidemiology, RNA vaccines

## Abstract

**Background:**

Nursing home (NH) residents have been severely affected during the COVID-19 pandemic because of their age and underlying comorbidities. Infection and outbreaks in NHs are most likely triggered by infected workers. Screening for asymptomatic NH workers can prevent risky contact and viral transmission to the residents. This study examined the effect of the BNT162b2 mRNA COVID‑19 (Comirnaty^®^; BioNTech and Pfizer) vaccination on the saliva excretion of SARS-CoV-2 among NH workers, through weekly saliva RT-qPCR testing.

**Methods:**

A 2-month cohort study was conducted among 99 NHs in the Walloon region (Belgium), at the start of February 2021. Three groups of workers, i.e., non-vaccinated (*n* = 1618), one-dosed vaccinated (*n* = 1454), and two-dosed vaccinated (*n* = 2379) of BNT162b2 mRNA COVID‑19 vaccine, were followed-up weekly. Their saliva samples were used to monitor the shedding of SARS-CoV-2. All positive samples were sequenced and genotyped to identify the circulating wild-type virus or variants of concern.

**Results:**

The protection fraction against the excretion of the SARS-CoV-2 in the saliva samples of the workers after the second dose is estimated at 0.90 (95% CI: 0.18; 0.99) at 1 week and 0.83 (95% CI: 0.54; 0.95) at 8 weeks. We observe more circulating SARS-CoV-2 and a greater variability of viral loads in the unvaccinated group compared to those of the vaccinated group.

**Conclusions:**

This field cohort study advances our knowledge of the efficacy of the mRNA BNT162b2 COVID-19 vaccine on the viral shedding in the saliva specimens of vaccinated NH workers, contributing to better decision-making in public health interventions and management.

## Plain language summary

Nursing homes have been particularly affected by COVID-19 outbreaks with devastating consequences. Screening for SARS-CoV-2 infection in nursing home workers is therefore helpful to prevent transmission of the virus. It is also helpful in determining whether vaccination, which has been widely implemented in this population, is effective at reducing the number of SARS-CoV-2 infections. To this end, we tested saliva samples from workers from 99 nursing homes in the Walloon region of Belgium over a two-month period. Some workers had not been vaccinated and others had received one or two doses of the Pfizer-BioNTech BNT162b2 vaccine. We find that fully vaccinated individuals are significantly protected against SARS-CoV-2 infection compared to non-vaccinated individuals. These findings help to provide evidence that BNT162b2 vaccination is an effective measure to limit the infection of nursing home workers.

## Abstract (web summary)

Saegerman et al. perform saliva SARS-CoV-2 testing in a cohort of nursing home workers in Belgium who are either unvaccinated or have received one or two doses of the BNT162b2 mRNA vaccine. The authors show that vaccination protects against shedding of SARS-CoV-2 into saliva and observe greater variability in viral load in the unvaccinated group.

## Introduction

Infection with Severe Acute Respiratory Syndrome Coronavirus 2 (SARS-CoV-2) induces coronavirus infectious disease 19 (COVID-19)^1^. The main routes include the direct contact and human-to-human transmission by infectious droplets or aerosol^2–4^.

Asymptomatic infection has been documented in several settings including in nursing homes (NHs)^5^. The proportion of asymptomatic infections varies widely between studies (from 18% to 62%). A meta-analysis reported that 20% of people remain asymptomatic throughout the course of infection^6^. In addition, a recent population study highlighted that asymptomatic individuals represent a significant risk for transmission of SARS-CoV-2^7^.

Due to the above-mentioned transmission characteristics, the SARS-CoV-2 has spread rapidly throughout China and the world since its first report on 31 December 2019^8^. Indeed, at the end of June 2021, COVID-19 resulted in high morbidity with more than 182 million confirmed cases worldwide^9^ and around 1.1 million in Belgium. In the meantime, there were more than 25.200 deaths in Belgium with 51.2% of these occurring in NH residents^10^. Therefore, NH residents have been severely affected by the COVID-19 pandemic. Infections of these people were possibly caused by infected workers in their direct surrounding environment^11^.

In NHs, workers who are asymptomatic SARS-CoV-2 carriers are most likely a source of contamination for residents, especially when visits of residents are suspended or strictly limited^7^. Therefore, screening for asymptomatic workers in NHs can prevent risky contact and viral transmission to elderly residents^4,11^.

Primary mitigation measures consisted of sanitary measures, i.e., compliance to strict hygiene protocols, societal protective measures like face mask wearing, physical distancing, proper room ventilation, and medical measures (i.e., vaccination)^4^. In Belgium, NHs were completely closed for visitors during the quarantine periods, or the number of visitors was highly restricted^12^, meaning that NH workers were the mere point of contact.

Since early 2021, vaccination has become a prominent management option to counter the COVID-19 pandemic^13^. Several vaccines were developed in a short time by making use of attenuated and inactivated viruses, viral vectors, nucleic acids, and proteins (for a review, see^14^), all of which have demonstrated a relative low rate of vaccine-related serious adverse events^13^. At the end of June 2021, more than 2.950 billion of vaccine doses had been administrated worldwide^9^. In the meantime, in Belgium, 10.9 million doses have been administered with some 4.1 million people being fully vaccinated out of a population estimated at 11.5 million^4^. Different marketed vaccines were successively authorised in Belgium, namely, vaccine Comirnaty^®^ (Pfizer/BioNTech) since December 21, 2020, vaccine Spikevax^®^ (Moderna) January 6, 2021, vaccine Vaxzevria^®^ (AstraZeneca) January 29, 2021, and finally, vaccine Janssen^®^ (Johnson & Johnson) March 11, 2021. The vaccination protocol for the first three vaccines consists of two doses, whereas only one dose is required for the last vaccine authorised. Since December 28, 2020 successive priority groups were vaccinated: since January 2021, residents and workers of NHs and health homes, as well as care workers in hospitals (doctors, nurses, etc.); since February 2021, first line health care staff (doctors, pharmacists, etc.) and staff from collective care institutions (e.g. care for people with disabilities) and other hospital staff; since March 2021, everyone from or older than 65 years; since April and May 2021, people at risk due to comorbidities, critical functions (e.g. police), pregnant womens, prison officers and (para)olympic athletes; and since June 2021, the rest of the general population from the age of 12 years. Initially limited to adults aged 18 years and above, the administration of the vaccines Comirnaty® and Spikevax^®^ was gradually opened up to young people from the age of 12 years.

In this regard, vaccination of NH workers is an additional medical preventive option. However, currently, the efficacy of the vaccination of NH workers related to the shedding of SARS-CoV-2 has been scarcely investigated.

The objective of this field cohort study was to assess how vaccination of NH workers could reduce asymptomatic cases of the SARS-CoV-2, which were detected by RT-qPCR assay using saliva specimens. Our results evidenced a significant reduction of asymptomatic cases of the SARS-CoV-2 among BNT162b2 mRNA COVID‑19 vaccinated workers and suggested the efficacy of the vaccination of workers in preventing viral transmission among NH residents.

## Methods

### Study population and timeline

Based on specific governmental measures implemented in Belgium during the COVID-19 pandemic, five periods of interest were identified: the first wave (starting from the documentation of the first infected case on March 2 until June 21, 2020), the inter-wave period (June 22 until August 30, 2020), the second wave (from August 31, 2020 until February 14, 2021), the third wave directly after the second (from February 15, 2021 until June 21, 2021), and the fourth wave starting at 4th October 2021^15^. The 2-month cohort study was conducted at the end of the second wave and the beginning of the third wave, from weeks 5 to 13 of 2021 (February and March 2021).

At the time of this study, there were 572 NHs active in the Walloon region of Belgium, 99 of which participated in the cohort study. In these 99 NHs, the number of residents was estimated at 7.651 individuals. Based on a ratio of 20.5 equivalent full-time workers for 30 residents^16^, the estimated study population of NH workers was around 5.228 adults.

### Vaccine used

The vaccination in the NHs was recommended by the Walloon Agency for a Quality Life (AViQ), on a voluntary basis. During the study period, only the BNT162b2 (Pfizer-BioNTech) COVID-19 vaccine was administered to the NH workers^17^. The vaccine was administrated intramuscularly in a two-dose regimen with a 3-week interval and indicated for active immunisation to prevent COVID-19 caused by SARS-CoV-2 virus, in individuals 12 years of age and older. In these populations, the estimated protection efficacy in preventing clinical disease is around 95%^17^ but does not reflect the efficacy towards virus shedding. The vaccine encodes a P2 mutant spike protein (PS 2) and is formulated as an RNA-lipid nanoparticle of nucleoside-modified mRNA^18,19^. In addition, the efficacy of m RNA BNT162b2 vaccine was estimated in field conditions as 95% (95% credibility interval: 90.3–97.6) in persons 16 years of age or older^20^.

### Experimental field design of the cohort study

**Fig. 1:**
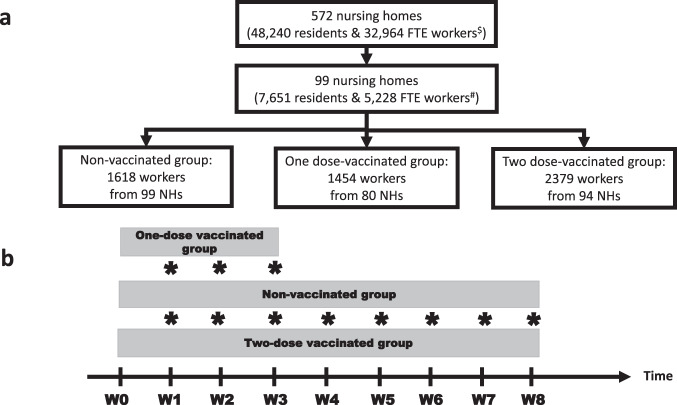
Experimental field design of the cohort study. **a** Study participants enroled. **b** Duration of the observation. ^$^The number of nursing homes (NHs), residents and workers were estimated in a previous study^21^. ^#^FTE, full-time equivalent. In the 99 studied NHs, the number of residents was estimated at 7.651 individuals. Based on a ratio of 20.5 equivalent full-time workers for 30 residents^16^, the estimated study population of NH workers was around 5228 adults. During the study, the maximum number of participating NH workers (i.e., physical persons; not all are employed full time) exceeded slightly the estimated FTE number by 4.38% (5457 *versus* 5228). *, points of comparison between each vaccinated group and the non-vaccinated group.

The experimental design of the cohort study was depicted in Fig. 1. Guidelines for reporting observational studies was followed (i.e. the Strengthening the Reporting of Observational Studies in Epidemiology—STROBE).

The starting date of the study was on the first sampling for the non-vaccinated group (NV group) and respectively, the date of the mRNA COVID-19 vaccine administration of the two vaccinated groups after getting the first dose (1D-V group) and the second dose (2D-V group). Within these conditions, point comparisons between the reference NV group with the 1D-V and 2D-V groups were performed on a weekly basis from week 1 to week 3 and from week 1 to week 8, respectively. All NH workers involved in the study presented no suspected symptoms of COVID-19 in the beginning of the study. We were not informed of any hospitalisation of one of the workers during the course of the study. The proportion of asymptomatic cases by group of workers (non-vaccinated, 1D-vaccinated, and 2D-vaccinated) followed the workflow of this study. Note that workers from the different groups were matched in the same NHs.

### Organisation of screening tests for NH workers

The logistical and organisational system, which aimed to guarantee a short sample delivery time, was based on eight fixed-relay points all over Walloon region. This system ensured a smooth workflow between NHs, suppliers, logistics operators, and the COVID-19 laboratory of the University of Liège.

### Saliva voluntary self-sampling kit

Fresh saliva was collected using a sampling device designed by the University of Liège and commercialised by Diagenode (Seraing, Belgium). The device does not require the intervention of medical personnel and contains an integrated process for viral inactivation. The self-sampling kit allowed for the collection of saliva by spitting. The kit was equipped with a dosing funnel that permitted the collection of exactly 1.2 mL of saliva which was subsequently mixed with 2 mL of lysis buffer. This buffer was developed during the design of the kit and contained a detergent and guanidium isothyocyanate. The mixing of the collected saliva with the buffer therefore inactivated any virus that might be present. The collection tubes were also heated in order to inactivate viral particles that might have been located on the outside of the tube or in the screw thread of the cap.

### Laboratory analysis

#### Ribonucleic acid (RNA) extraction from saliva and pooling

In the collection device, saliva was diluted to a 1:1 ratio with an extraction buffer containing 1 M guanidine thiocyanate (GITC). Samples were incubated at 80 °C for 40 min. The pooling RNA extraction procedure consisted in mixing 60 µL of saliva from three different workers with 180 µL of buffer containing GITC 4 M to obtain a total volume of 360 µL. For individual RNA extractions, 100 µL of saliva per sample was added to 300 µL of a lysis buffer containing GITC 4 M. All samples were spiked with a purified MS2 bacteriophage according to the manufacturer’s protocol (Thermo Fisher A47817). RNA extraction was performed using the CoRNA Isolation Kit (Diagenode, Seraing, Belgium) and 50 µL of magnetic beads. Extracted RNA was eluted from magnetic beads in 50 µl of UltraPure DNase/RNasefree distilled water.

#### RT-qPCR assay

We performed a multiplex RT-qPCR assay using the TaqPath RT-PCR COVID-19 kit (ThermoFisher A47817) together with the TaqPath one-step master mix – No ROX (ThermoFisher CN A28523). This RT-qPCR assay targets three viral genes, *ORF1ab*, *N* and *S* genes. All reactions were performed using a 384w format (final volume of 20 µL) in a QS5 thermocycler (Applied Biosciences, Waltham, Massachusetts, USA). RT-qPCR reactions were prepared as follows: 5 µL of 4× TaqPath Multiplex MasterMix, 1 µL of COVID-19 Real-Time PCR assay, 6 µL of water and 8 µL of RNA (samples or controls). TaqPath™ COVID-19 positive Control (ThermoFisher A48003, ThermoFisher, Waltham, Massachusetts, USA) at 25 genomic copies/µL was used. The RT-qPCR was run in standard mode, consisting of a hold stage at 25 °C for 2 min, 53 °C for 10 min, and 95 °C for 2 min, followed by 40 cycles of a PCR stage at 95 °C for 3 s, then 60 °C for 30 s with a 1.6 °C/s ramp up and down rate. Results were analysed using FastFinder software (Ugentec, Hasselt, Belgium) and expressed as quantification cycles (Cq value, i.e., number of cycles required for the quantification of a fluorescent signal to be considered positive) with a positivity limit fixed at a Cq <37.

During this cohort study, all RT-qPCR positive saliva samples were tested (i.e. initial individual sample) to obtain a genotype of the SARS-CoV-2 in order to assess the circulating strains (wild-type versus variants of the SARS-CoV-2). Using the equation of calibration curve, the number of genomic copies was estimated for each saliva sample of the RT-qPCR positive workers and for the gene *ORF1ab* and *N protein*.

#### Interpretation of results

Samples were considered uninterpretable when the MS2 internal control was over a Cq value of 30 (problem of RNA extraction). In reverse, the samples were interpretable and subsequently negative or positive when the viral genes detected were over or under a Cq value of 37, respectively.

All individual samples were pooled by three. If a pool was negative, the status of all associated individual samples was considered negative. If a pool was positive or inconclusive, each associated sample was retested individually. Pooling only induced a negligible decrease of sensitivity estimated at 0.33%^21^.

#### SARS-CoV-2 genotyping

SARS-CoV-2 RNA was reverse transcribed, amplified and S protein E484K, N501Y, K417N and K417T mutations were detected using Taqman 1-Step RT-QPCR master mix (A15299, Thermofisher) with probes and primers from Taqman SNP assays (4332075, Thermofisher, assay IDs: ANPRYZA for N501Y, ANU7GMZ for E84K, ANZTTXP for K417N, AN49ARF for K417T) following the manufacturer’s protocol. The real-time RT-QPCR was performed with a QuantStudio 5 real-time PCR instrument (Thermofisher) in genotyping mode and data analysed with the QuantStudio design and analysis software.

#### SARS-CoV-2 sequencing

RNA was treated with Chelex-100 prior to Reverse Transcription. 10 µl of sample were mixed with 2 µl of 10% (wt/vol) Chelex-100 (Bio-Rad Laboratories, Richmond, CA) in sterile water and incubated at 98 °C for 2 min. Samples were centrifuged and the supernatant collected. 8 µL of RNA were combined with 2 µl of SuperScript IV VILO Master Mix to carry out Reverse Transcription. This was incubated at 25 °C for 10 min, 50 °C for 10 min and 85 °C for 5 min. PCR was carried out using Q5^®^ High-Fidelity DNA Polymerase (NEB) using the version 3 ARTIC Network amplicons. PCR conditions followed the recommendations in the sequencing protocol of the ARTIC Network. We multiplexed the samples following the manufacturer’s recommendations using the Oxford Nanopore Native Barcoding Expansion 96 kit in conjunction with Ligation Sequencing Kit 109 (Oxford Nanopore). Sequencing was carried out on a Minion using R9.4.1 flow cells. Variant calling and consensus genomes were generated via the ARTIC Network bioinformatics protocol^22^.

### Statistics and reproducibility

Representativeness was checked by comparing characteristics (province and size of NH with interaction) of participating *versus* non-participating NHs using logistic regression models^23^. The Hosmer-Lemeshow test was used to assess the goodness-of-fit of the model^24^.

The incidence rate (IR) was defined as the number of newly positive NH workers (i.e., negative the week before) divided by the number of tested NH workers with conclusive results.

The incidence rate ratio (IRR) for a specific week was defined as the ratio between the IR of the vaccinated group and that of the non-vaccinated group. The cumulative IRR (cIRR) was defined as the IRR for a specific number of weeks. The protection fraction (PF) was defined as one minus the cIRR. Exact binomial distributions were used to derive the 95% confidence interval (95% CI) of the cIRR and the PF over time^24^.

For the comparison between non-vaccinated and 2D-vaccinated groups, the statistical power (i.e. 1−β with β, the false negative rate) was estimated using the command sampsi of Stata/SE 14.2 (StataCorp LLC, College Station, Texas, USA).

In addition, a sensitivity analysis was performed in order to verify if the above cIRR was not affected by the fluctuation of the sampling effort as a function of time. Accordingly, the obtained estimate was compared with the results achieved from ten bootstraps of respectively 1000 and 800 weekly samples from non-vaccinated and 2D-vaccinated groups of NH workers.

The normality of the distribution of genomic copies (g.c.) and the logarithm of g.c. for both *ORF1ab* gene and *N protein* gene was tested using a Shapiro-Wilk W test. The logarithm of the number of genomic copies present in the saliva of positive samples from workers depending of the vaccine status and in function of wild-type virus and alpha variant was compared using a linear regression^24^. The variability of the number of g.c. was assessed calculating its interquartile range (IQR), i.e, the difference between the 1st and 3rd quartiles and encloses the central 50% of the observations if the observations are arranged in rank order^24^. Indeed, the IQR represents the non-parametric variability of a dataset. The IQR is influenced neither by the presence of outliers nor by the sample size, which justified the reason for its use. The comparison between frequency detection of S protein in wild-type virus and alpha variant was assessed using a Firth logit regression^23^.

All analyses were performed using Stata SE 14.2 (StataCorp, College Station, Texas, USA). The significance level was set to 0.05. In addition, Quantum GIS (Geographic Information System) version 3.16.2 was used to construct specific maps.

### Blinding

Because of the nature of this field cohort study, no intervention on the choice of vaccination (non-vaccinated, 1D-vaccinated and 2D-vaccinated) was made for each participant. The sampling system was fully designed to allow anonymity of individual results. The only link between a sample and a test result was the barcode number on the saliva sample tube. The barcodes of the saliva samples of a nursing home were scanned at the time of submission to ensure traceability of the results. On the basis of this operation, it was possible to match anonymous saliva results with a specific nursing home, and thus obtain information on the positivity rate within this establishment. Scanning the barcodes did not permit identifying the person to whom a sample belonged.

## Results

### Representativeness

**Fig. 2:**
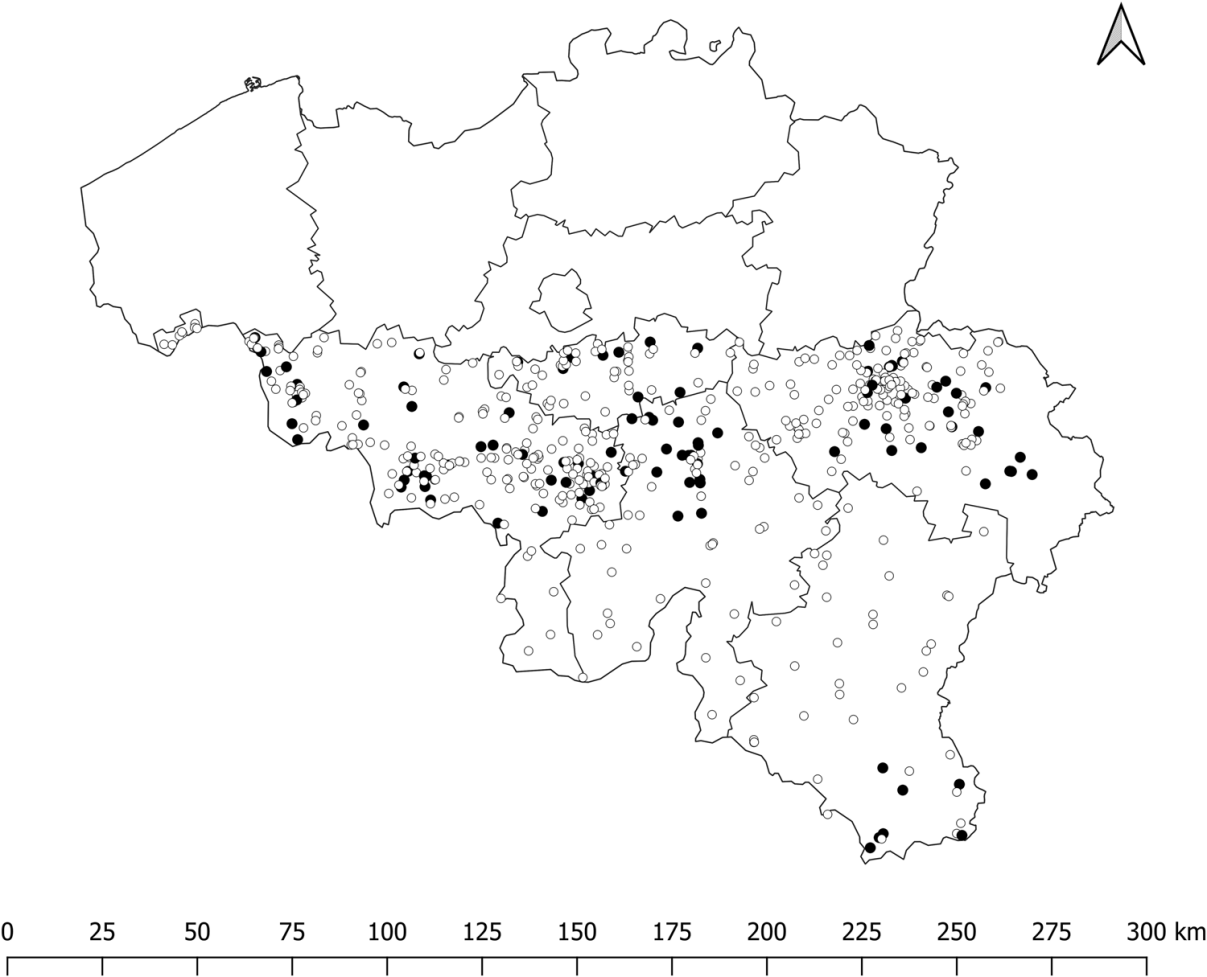
Participating (*N* = 99) and non-participating (*N* = 472) nursing homes in the study; map of Belgium, with Wallonia located in the south. Black circles, participating nursing homes; White circles, non-participating nursing homes.

In a previous study, the number of active NHs in the Walloon region of Belgium was estimated at 572^21^. Based on this sampling frame, no significant difference was observed between participating (*N* = 99) and non-participating (*N* = 472) NHs in the cohort study according to province and NH worker population size taking into account the possible interaction between the two parameters (Logistic regression; *p* value > 0.10) (Fig. 2). The chi-square of the Hosmer-Lemeshow goodness-of-fit test (8 df; *α* = 0.05) was equal to 3.73 (*p* value = 0.88), as a consequence, NH representativeness was considered acceptable.

### Sampling effort

**Fig. 3:**
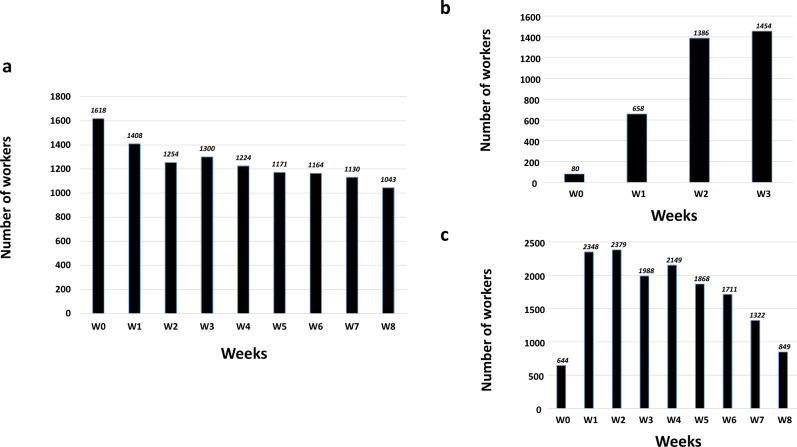
Sampling effort by week for each group of nursing home workers. **a** Non-vaccinated group of workers (from 99 nursing homes). **b** On dose-vaccinated group of workers (from 80 nursing homes). **c** Two dose-vaccinated group of workers (form 94 nursing homes). The maximum numbers of workers for each group (1618 + 1454 + 2379 = 5451) were close to the 100% of eligible workers (5228). The slight difference is related to the fact that some workers are not fully-employed.

The number of NH workers (i.e., those who performed the saliva test) involved in the cohort study over time is depicted in Fig. 3 and Supplementary Data 1. Vaccinated (1D-V and 2D-V groups of NH workers) and non-vaccinated NH workers originating from 80, 94 and 99 NHs, respectively. Indeed, most of the workers of the three groups were originating from the majority of the NHs initially included in the cohort study, rendering a comparable exposure probability to SARS-CoV-2 (Supplementary Fig. 1).

### Cumulative incidence rate ratio

The incidence rate ratio (IRR) was estimated from week 1 up to week 3 for the 1D-V group of NH workers and from week 1 up to week 8 for the 2D-V group of NH workers (Fig. 1). As a reminder, the cumulative incidence rate ratio (cIRR) was estimated over time for both 1D-V group and 2D-V group considering the NV group as the reference group (Table 1). No significant cIRR was observed after the three first week after getting the first shot of vaccine. However, significant cIRR and associated protection fraction (PF = 1 − cIRR) were observed each week during the 8 week-period after getting the second shot of vaccine. The PF ranges between 0.90 (95% CI: 0.18-0.99) and 0.83 (95% CI: 0.54–0.95) after one and eight weeks, respectively. Indeed, after 8 weeks, 20 cases for non-vaccinated were counted and only 5 who have received at least 2 doses of the vaccine. This difference was significant (Fisher’exact test, *p* value < 0.001). Considering the smallest or the highest monthly sample size of workers counted (worst- and best-case scenarios), the estimated statistical power for two-sample comparison of proportions (in 2D-vaccinated group versus non-vaccinated group) was between 0.65 and 0.97. A power of 0.95 was yielded if we considered the monthly average sample size of workers observed.

**Table 1:** Estimation of the cumulative incidence rate ratio and the related protection fraction against excretion of SARS-CoV-2 in saliva samples of vaccinated nursing home workers with COVID-19 mRNA BNT162b2 vaccine.

Comparison between the one-dose vaccinated workers and the non-vaccinated workers								
Group	Week	Np	cNp [A]	Nt	cNt [B]	IR [A] / [B]	cIRR (95% CI) [C]	PF (95% CI) 1 – [C]
NV	W1	6	6	1408	1408	0.0043	-	-
	W2	1	7	1254	2662	0.0026	-	-
	W3	1	8	1300	3962	0.0020	-	-
1D-V	W1	1	1	658	658	0.0015	0.3566 (0.0078–2.9395)	nd
	W2	1	2	1386	2044	0.0010	0.3721 (0.0377–1.9543)	nd
	W3	0	2	1454	3498	0.0006	0.2832 (0.0293–1.4189)	nd
Comparison between the two-dose vaccinated workers and the non-vaccinated workers								
Group	Week	Np	cNp [A]	Nt	cNt [B]	IR [A] / [B]	cIRR (95% CI) [C]	PF (95% CI) 1 – [C]
NV	W1	6	6	1408	1408	0.0043	-	-
	W2	1	7	1254	2662	0.0026	-	-
	W3	1	8	1300	3962	0.0020	-	-
	W4	2	10	1224	5186	0.0019	-	-
	W5	3	13	1171	6357	0.0020	-	-
	W6	3	16	1164	7521	0.0021	-	-
	W7	3	19	1130	8651	0.0022	-	-
	W8	1	20	1043	9694	0.0021	-	-
2D-V	W1	1	1	2348	2348	0.0004	0.0999 (0.0022–0.8238)	0.9001 (0.1762–0.9978)
	W2	0	1	2379	4727	0.0002	0.0804 (0.0018–0.6262)	0.9196 (0.3738–0.9982)
	W3	1	2	1988	6715	0.0003	0.1475 (0.0153–0.7391)	0.8525 (0.2609–0.9847)
	W4	0	2	2149	8864	0.0002	0.1170 (0.0125–0.5491)	0.8830 (0.4509–0.9875)
	W5	1	3	1868	10732	0.0003	0.1367 (0.0250–0.4974)	0.8633 (0.5026–0.9750)
	W6	1	4	1711	12443	0.0003	0.1511 (0.0368–0.4684)	0.8489 (0.5316–0.9632)
	W7	0	4	1322	13765	0.0003	0.1323 (0.0327–0.3981)	0.8677 (0.6019–0.9673)
	W8	1	5	849	14614	0.0003	0.1658 (0.0486–0.4553)	0.8342 (0.5447–0.9514)

*NV* non-vaccinated workers, *1D-V* one-dose vaccinated workers, *2D-V* two-dose vaccinated workers, *Np* number of positive RT-qPCR, *cNp* cumulated Np, *Nt* number of tested NH workers with RT-qPCR, *cNt* cumulated Nt, *IR* incidence rate, *cIRR* cumulative incidence rate ratio, *PF* protection fraction of workers against excretion of SARS-CoV-2 in saliva samples, *CI* Exact binomial approximation of the confidence interval, *nd* not determined because the cIRR is not significant.

### Sensitivity analysis

**Fig. 4:**
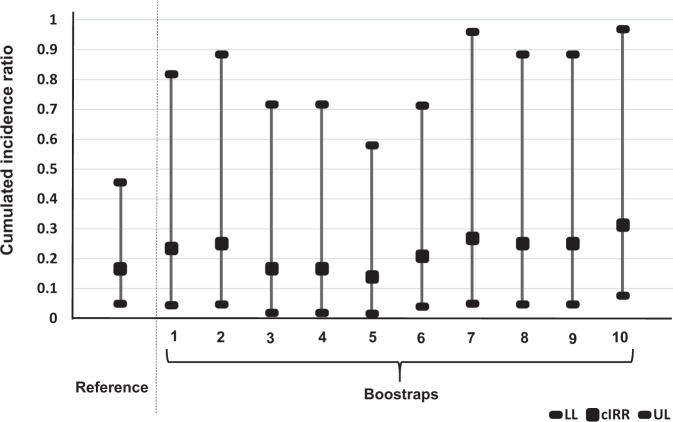
Sensitivity analysis based on bootstraps estimating the cumulative incidence rate ratio of excretion of SARS-CoV-2 in saliva sample of fully vaccinated workers from nursing homes with COVID-19 mRNA BNT162b2 vaccine. cIRR, cumulative incidence rate ratio; LL and UL are the lower and the upper limits of the 95% confidence interval (exact binomial approximation).

In order to verify if the estimation of the cIRR and the related PF against excretion of SARS-CoV-2 was not affected by the fluctuation of the sampling effort over time, a sensitivity analysis was performed using ten bootstraps of 1000, and 800 samples from the NV and 2D-V groups of workers, respectively (in each week). The estimated cIRR from each bootstrap was depicted in Fig. 4 and Supplementary Data 2. The estimated cIRR obtained using all data (Table 1) was used as reference point. No significant difference was observed between the reference point and the different bootstraps (based on the 95% CI overlapping). In addition, the upper of all 95% CI was below one that confirms in all cases a significant protection of 2D-V group against excretion of SARS-CoV-2.

### Circulating virus strain

**Fig. 5:**
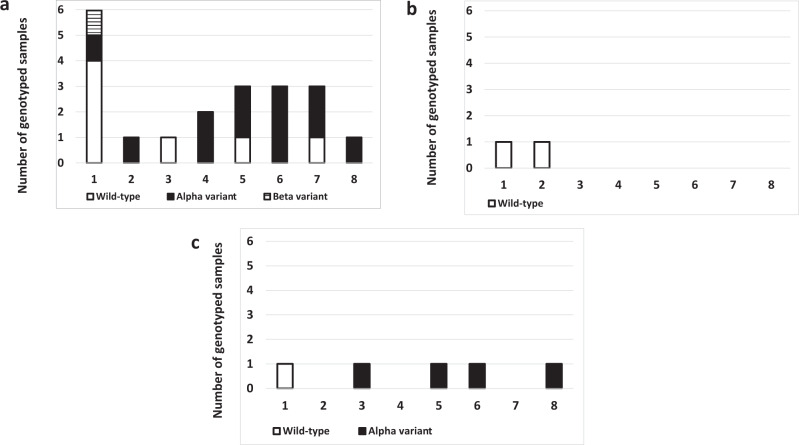
Number of wild-type virus and variants of concern identified over time in function of the vaccine status. **a** Non-vaccinated workers. **b** One-dose vaccinated workers. **c** Two-dose vaccinated workers. Legend: W, week.

All samples that were positive by RT-qPCR (*n* = 27) were sequenced and submitted to genotyping. Among those samples, 23 gave conclusive results both at genotyping and sequencing, two samples gave only a conclusive result at genotyping and two sample gave only a conclusive result at sequencing. Among the samples that gave a conclusive result either by genotyping or sequencing (*n* = 27), the proportion of variants of concern and wild-type virus was not dependent on the status of worker, i.e. vaccinated versus non-vaccinated (Fisher’s exact test; *p* value = 0.18). The circulating genotypes over time identified were presented, separately for the three groups of workers, in Fig. 5 and Supplementary Data 3. Higher levels of detection of circulating virus strains were observed in the non-vaccinated group of workers (*N* = 20; 74.1% with 95% CI: 53.7–88.9) in comparison with both 1D-vaccinated (*N* = 2; 7.4% with 95% CI: 0.9–2.4) and 2D-vaccinated (*N* = 5; 18.5 with 95% CI: 6.3–38.1) groups of workers.

### Estimation of the genomic copies in saliva samples of the RT-qPCR positive workers

**Fig. 6:**
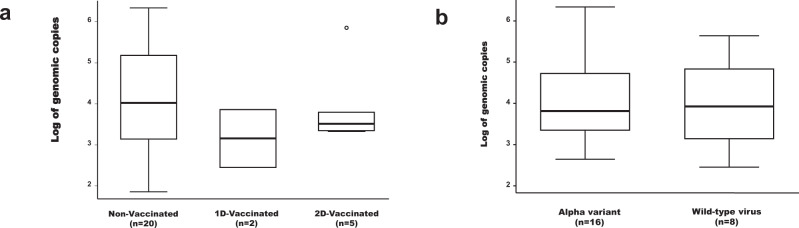
Logarithm of the number of genomic copies estimated based on ORF1ab gene. **a** Non-vaccinated *versus* vaccinated groups. **b** Wild-type virus *versus* Alpha variant. Log, logarithm in base ten. The dashed line represents the median of the log of genome copies; the solid lines below and above each rectangle represent, respectively, the first and the third quartiles; adjacent lines to the whiskers represent the limits of the 95% confidence interval; small circles represent outside values.

The number of g.c. was not normally distributed for both *ORF1ab* gene and *N protein* gene (Shapiro-Wilk W test; *p* value < 0.0001) but well after logarithmic transformation (Shapiro-Wilk W test; *p* value > 0.10). A good correlation was observed between the logarithm of the number of g.c. using *ORF1ab* and *N protein* (Person coefficient correlation = 0.984; *p* value < 0.0001). No statistical difference in the number of g.c. for both *ORF1ab* gene (Linear regression; *p* value > 0.34) and for *N protein* gene (Linear regression; *p* value > 0.37) was observed between the three groups of workers (i.e., NV, 1D-V and 2D-V) (Fig. 6A). Using a semi-quantitative recommended classification of the number of g.c. as weak positive (<10^3 ^g.c.), moderate positive (10^3^–10^5^ g.c.), strongly positive (10^5^–10^7 ^g.c.) and very strongly positive (>10^7 ^g.c.)^25^, and grouping, in one side, weak and moderate positive and in other side, strongly and very strongly positive, no statistical difference between the three groups in the number of g.c. for both *ORF1ab* gene (Fisher’s exact test; *p* value = 0.33) and for *N protein* gene (Fisher’s exact test; *p* value = 0.24) was observed. However, the interquartile range of g.c. (IQR) was higher in non-vaccinated group of workers (IQR_*ORF1ab*_ = 143,717; IQR_*N protein*_ = 52,219) in comparison with both 1D-vaccinated (IQR_*ORF1ab*_ = 3487; IQR_*N protein*_ = 1493) and 2D-vaccinated (IQR_*ORF1ab*_ = 4044; IQR_*N protein*_ = 7882) groups of workers.

The comparison of saliva samples in which wild-type virus or alpha variant were identified indicated no statistical difference in the logarithm of g.c. for both *ORF1ab* gene (Linear regression; *p* value = 0.83) and for *N protein* gene (Linear regression; *p* value = 0.26) (Fig. 6B).

In addition, the S protein gene was undetected in 20 saliva samples among the 27 tested for genotyping (74%; 95% CI: 54–89). Among the 20 positive samples that had a S dropout, only 16 were genotyped as Alpha variant. The four remaining contained too low genomes to allow us to genotype them. As the S PCR of our triplex PCR was the least sensitive, we could not distinguish between any variant. Using wild-type virus as the reference group, the absence of the detection of S protein was statistically related to the alpha variant (Firth logit regression; OR = 86; 95% CI: 4–2040; *p* value = 0.006).

## Discussion

This field cohort study examined the effect of the BNT162b2 mRNA COVID‑19 (Comirnaty^®^; BioNTech and Pfizer) vaccination of NH workers, on their saliva excretion of the SARS-CoV-2 using weekly saliva RT-qPCR testing. Main results indicated a significant and relevant reduction of excretion of the SARS-CoV-2 in the fully vaccinated group compared to the reference non-vaccinated group. No significant protection could be documented after the workers received the first dose of the mRNA COVID-19 vaccine (3-week follow up), but only 1-week after the administration of the second dose of the same vaccine. Indeed, after the second dose of the vaccine administration, the protection fraction against the excretion of the SARS-CoV-2 in saliva samples of workers was estimated at 0.90 (95% CI: 0.18; 0.99) and 0.83 (95% CI: 0.54; 0.95) after one and eight weeks, respectively. Considering the fact that 20% of people (i.e., most likely value and with range from 18% up to 62%) remain asymptomatic throughout the course of infection^6^ and represent a significant risk for transmission of SARS-CoV-2^7^, this protection fraction could reduce transmission from asymptomatic workers to NH residents.

After 8 weeks, a significant difference was counted in the number of cases (20 for non-vaccinated versus 5 for 2-D vaccinated workers) with an estimated statistical power of 0.65, 0.97 and 0.95 considering the smallest, the highest and the average monthly sample size of workers, respectively. As the minimum of 80% is often considered conventional in biomedical sciences^26^, the statistical power of this study should be considered as acceptable.

Saliva collection is considered as a potential alternative to nasopharyngeal sampling because of its technical simplicity^21^, which is of interest for weekly repeated sampling strategies^27^. Moreover, that higher viral load in saliva samples is associated with COVID-19 symptomatic cases and oral cavity as an important site for SARS-CoV-2 infection makes saliva a potential route of SARS-CoV-2 transmission^28^. According to a recent meta-analysis, the sensitivity of the saliva RT-qPCR in detecting SARS-CoV-2 was 91% (95% CI: 80–99), compared to 98% (95% CI: 89–100) for nasopharyngeal swabs (NPS)^27^. Another review reported a non-significant difference in viral loads between nasopharyngeal or sputum and saliva samples^29^. However, greater variation of viral loads has been observed with NPS compared to those from saliva specimens^30^. In a cohort study of asymptomatic health care workers (*n* = 493), 13 tested positive for SARS-CoV-2 through saliva testing. Nine of these were also tested through NPS sampling, of which seven were negative. All 13 health care workers who tested positive through saliva testing were re-confirmed to be infected^30^.

In addition, the ability to sequence SARS-CoV-2 RNA from saliva was previously evidenced^31^. Finally, the pooling of samples (i.e., testing a few samples at once) may allow for the detection of SARS-Cov-2 with sufficient diagnostic accuracy^32–34^. Previous studies in NH workers employing the same methodology as the present cohort study have estimated a reduction of sensitivity at 0.3% due to the pooling (i.e., testing three samples at once)^21^.

Currently, the WHO has defined four variants of concern (VOC) with multiple substitutions in the spike protein (Table 2). The qualification of VOC is related to public health significance with the inclusion of one or more of the following criteria: increase in transmissibility or detrimental change in COVID-19 epidemiology; or increase in virulence or change in clinical disease presentation; or decrease in effectiveness of public health and social measures or available diagnostics, vaccines, or therapeutics. In addition, the WHO defined variants of interest (VOI), with genome mutations established or suspected to induce phenotypic implications (i.e. community transmission/multiple COVID-19 cases/clusters, or has been detected in multiple countries; or assessed as VOI by the WHO ad hoc working group on SARS-CoV-2 virus evolution) (https://www.who.int/en/activities/tracking-SARS-CoV-2-variants/).

**Table 2:** List of variants of concern according to the WHO (situation as of November 2021).

WHO label	Pango lineage	GISAID clade/lineage	Next strain clade	Earliest documented samples	Date of designation
Alpha	B.1.1.7	GRY (formerly GR/501Y.V1)	20I (V1)	United Kingdom, Sep-2020	18-Dec-2020
Beta	B.1.351	GH/501Y.V2	20H (V2)	South Africa, May-2020	18-Dec-2020
Gamma	P.1	GR/501Y.V3	20J (V3)	Brazil, Nov-2020	11-Jan-2021
Delta	B.1.617.2	G/478K.V1	21A, 21I, 21J	India, Oct-2020	VOI: 4-Apr-2021 VOC: 11-May-2021
Omicron	B.1.1.529	GR/484A	21K	Multiples countries	VUM: 24-Nov-2021 VOC: 26-Nov-2021

During this field cohort study, all positive RT-qPCR samples were sequenced and genotyped to identify the SARS-CoV-2 circulating strain (Fig. 5 and Supplementary Data 3). This protocol could be of interest to investigate the efficacy of the vaccination protocol against these circulating viral strains, which keep evolving. In this study, more numerous circulating strains of virus were observed in non-vaccinated group of workers in comparison with vaccinated groups of workers. As there are fewer infections in the vaccinated groups of NH workers, there was less detection of variants. In addition, the fast rate of vaccination decreased the probability of occurrence of resistant strains^35^.

The saliva sampling device used in this study contained an integrated process for viral inactivation (i.e. biosafety reason) that did not permit the dosage of IgA^21^. However, the added-value of the dosage of IgA merits consideration for future research because neutralising IgA remained detectable in saliva samples for a longer time (until 49 to 73 days of post-symptoms) than in serum (decreased notably one month after the onset of symptoms)^36^. In addition, recent preliminary studies indicated the elicitation of neutralising antibodies by COVID-19 mRNA vaccine against both wild-type and at least three SARS-CoV-2 variants^17,37,38^ but less against variant delta^39^. Indeed, both RT-qPCR and detection of IgA antibodies in saliva samples could be a good strategy to monitor the effect of the vaccine on the circulating SARS-CoV-2 strains (wild-type virus and variants).

In this cohort study, the number of genomic copies was not significantly different between non-vaccinated, 1D-vaccinated and 2D-vaccinated groups of workers, both for *ORF1ab* and for *N protein* genes. However, the IQR of the g.c. was more important in the non-vaccinated group of workers in comparison with vaccinated workers for both *ORF1ab* and for *N protein* genes indicating a wider variability of the viral load in the non-vaccinated group of workers. Recent reports indicated a reduction of the SARS-CoV-2 viral load after administration of the BNT162b2 vaccine in health care workers contributing to a lower virus spread^40,41^.

Moreover, the number of genomic copies for two different genes (*ORF1ab* and *N protein*) was not different between wild-type and alpha variant (Fig. 6 and Supplementary Data 4). Indeed, the viral load does not seem to be related to the virulence of the genotype studied. However, the number of samples included in this comparison was relatively small. Therefore, further investigation is needed.

In addition, as expected, the absence of the detection of S protein was significantly related to the alpha variant in comparison with the wild-type virus. The monitoring over time of this detection failure should indicate the presence of variant(s).

Some limitations are inherent in this field cohort study as the probability of individual exposure to SARS-CoV-2 virus of individual NH workers can be related to the local basic reproductive number (R0). However, in the absence of availability of this local R0, the trend of the R0 in function of province over time was monitored during the study. No particular difference was observed in this trend according to the province but well over time (Supplementary Fig. 1). Indeed, the assumption of the homogeneity of the exposure between provinces could be considered acceptable. In addition, the observed difference of R0 over time (whatever the province considered) can be a plausible explanation of some limited fluctuations in the cIRR and associated PF in function of weeks after the complete primo-vaccination. Another limitation is related to the fluctuation of the weekly participation rate of NH workers (Fig. 3 and Supplementary Data 1). In order to test if this fluctuation had certain effect on the results obtained, a sensitivity analysis was performed using bootstrapping method. Accordingly, no particular detrimental effect was documented, which further confirmed the robustness of the results.

In conclusion, weekly saliva RT-qPCR testing for SARS-CoV-2 demonstrated a significant effect of the COVID-19 mRNA BNT162b2 vaccine on the viral shedding in the saliva samples of vaccinated NH workers. This vaccination contributes to disrupting the chain of transmission of SARS-CoV-2 between NH workers and NH residents. Field follow-up of vaccinated cohorts concerning both viral excretion and identification of circulating SARS-CoV-2 strains are of prime importance to properly assess the effectiveness of the vaccination protocol on wild-type virus and its variants. IgA antibodies saliva titration should be considered in similar protocols in order to test their neutralisation capacity against the circulating SARS-CoV-2 strains. Such monitoring should advance our knowledge of the efficacy of the currently approved COVID-19 vaccines and immunological responses, hence contributing to better decision-making in public health interventions and management.

### Reporting summary

Further information on research design is available in the Nature Research Reporting Summary linked to this article.

## Supplementary information

Supplementary Information

Supplementary Data 1

Supplementary Data 2

Supplementary Data 3

Supplementary Data 4

Reporting Summary

## Data Availability

The data that support the findings of this study are available from the corresponding author upon request. Source data for Figs. 3 –6 in the manuscript is available as Supplementary Data 1, 2, 3 and 4, respectively. Complete SARS-CoV-2 genome sequences have been deposited in GISAID with accession numbers EPI_ISL_7017971 to EPI_ISL_7017981. Partial sequences have been deposit in ENA with the study accession ID PRJEB49173.
