# Supplementary material for: A 2-month field cohort study of SARS-CoV-2 in saliva of BNT162b2 vaccinated nursing home workers
Source: Commun Med (Lond). 2022 Jan 10;2:1. doi: 10.1038/s43856-021-00067-3 (PMC9053279; doi:10.1038/s43856-021-00067-3)
Supplement: Supplementary file 1 — Supplementary Information [file 43856_2021_67_MOESM1_ESM.pdf]

**Supplementary Figure 1.** Trends observation of the basic reproduction number in function of province over time (Source: https://covid-19.sciensano.be/fr/covid-19-situation-epidemiologique).

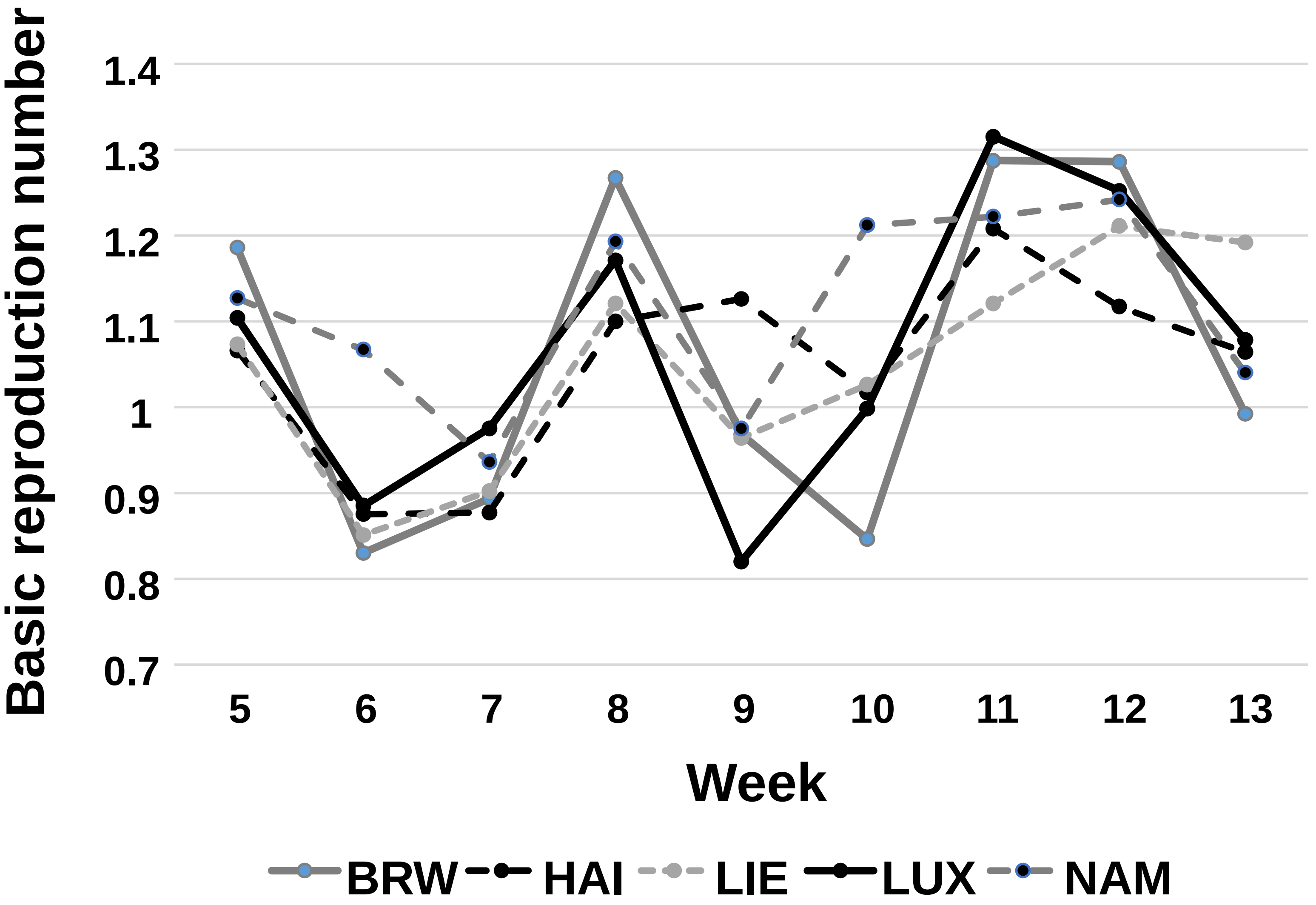


Legend: BRW, Walloon Brabant; HAI, Hainaut; LIE, Liège; LUX, Luxembourg; NAM, Namur (i.e. different provinces of the Walloon Region).
