# Supplementary material for: A 2-month field cohort study of SARS-CoV-2 in saliva of BNT162b2 vaccinated nursing home workers
Source: Commun Med (Lond). 2022 Jan 10;2:1. doi: 10.1038/s43856-021-00067-3 (PMC9053279; doi:10.1038/s43856-021-00067-3)
Supplement: Supplementary file 6 — Reporting Summary [file 43856_2021_67_MOESM6_ESM.pdf]

nature research

Corresponding author(s): Prof. Claude Saegerman

Last updated by author(s): Dec 4, 2021

Reporting Summary

Nature Research wishes to improve the reproducibility of the work that we publish. This form provides structure for consistency and transparency in reporting. For further information on Nature Research policies, see our Editorial Policies and the Editorial Policy Checklist.

Statistics

For all statistical analyses, confirm that the following items are present in the figure legend, table legend, main text, or Methods section.

n/a	Confirmed	
☐	☒	The exact sample size (*n*) for each experimental group/condition, given as a discrete number and unit of measurement
☐	☒	A statement on whether measurements were taken from distinct samples or whether the same sample was measured repeatedly
☐	☒	The statistical test(s) used AND whether they are one- or two-sided<br>*Only common tests should be described solely by name; describe more complex techniques in the Methods section.*
☐	☒	A description of all covariates tested
☐	☒	A description of any assumptions or corrections, such as tests of normality and adjustment for multiple comparisons
☐	☒	A full description of the statistical parameters including central tendency (e.g. means) or other basic estimates (e.g. regression coefficient) AND variation (e.g. standard deviation) or associated estimates of uncertainty (e.g. confidence intervals)
☐	☒	For null hypothesis testing, the test statistic (e.g. *F*, *t*, *r*) with confidence intervals, effect sizes, degrees of freedom and *P* value noted<br>*Give P values as exact values whenever suitable.*
☒	☐	For Bayesian analysis, information on the choice of priors and Markov chain Monte Carlo settings
☒	☐	For hierarchical and complex designs, identification of the appropriate level for tests and full reporting of outcomes
☒	☐	Estimates of effect sizes (e.g. Cohen's *d*, Pearson's *r*), indicating how they were calculated

Our web collection on statistics for biologists contains articles on many of the points above.

Software and code

Policy information about availability of computer code

Data collection	Collection and Analysis of Data and Information of Strategic Utility (acronym: RADIUS), Liège University, Liège, Belgium
Data analysis	All analyses were performed using Stata SE 14.2 (StataCorp, College Station, Texas, USA). I<br>n addition, Quantum GIS (Geographic Information System) version 3.16.2 was used to construct specific maps.

For manuscripts utilizing custom algorithms or software that are central to the research but not yet described in published literature, software must be made available to editors and reviewers. We strongly encourage code deposition in a community repository (e.g. GitHub). See the Nature Research guidelines for submitting code & software for further information.

Data

Policy information about availability of data

All manuscripts must include a data availability statement. This statement should provide the following information, where applicable:
- Accession codes, unique identifiers, or web links for publicly available datasets
- A list of figures that have associated raw data
- A description of any restrictions on data availability

The data that support the findings of this study are available from the corresponding author upon request. Source data for figures 3 to 6 in the manuscript is available as Supplementary Data 1, 2, 3 and 4, respectively.

Field-specific reporting

Please select the one below that is the best fit for your research. If you are not sure, read the appropriate sections before making your selection.

☒ Life sciences ☐ Behavioural & social sciences ☐ Ecological, evolutionary & environmental sciences

For a reference copy of the document with all sections, see nature.com/documents/nr-reporting-summary-flat.pdf

Life sciences study design

All studies must disclose on these points even when the disclosure is negative.

Sample size	At the time of this study, there were 572 nursing homes active in the Walloon region of Belgium, 99 of which participated in the cohort study (voluntary basis). In these 99 nursing homes, the number of residents was estimated at 7651 individuals. Based on a ratio of 20.5 equivalent full-time workers for 30 residents [Rombeaux JM, 2017], the estimated study population of nursing home workers was around 5228 adults. Representativeness was checked by comparing characteristics (province and size of nursing homes with interaction) of participating versus non-participating ursing homes using Firth's logistic regression models [Heinze & Schemper, 2002]. The Hosmer-Lemeshow test was used to assess the goodness-of-fit of the model [Petrie and Watson, 2013].
Data exclusions	No data were excluded.
Replication	A sensitivity analysis was performed in order to verify if the cumulate incidence rate ratio was not affected by the fluctuation of the sampling effort as a function of time. Accordingly, the obtained estimate was compared with the results achieved from ten bootstraps of respectively 1000 and 800 weekly samples from non-vaccinated and 2Doses-vaccinated groups of nursing home workers.
Randomization	Vaccinated (1Dose-Vaccinated and 2doses-Vaccinated groups of nursing home workers) and non-vaccinated nursing home workers originating from 80, 94 and 99 nursing homes, respectively. Indeed, most of the workers of the three groups were originating from the majority of the nursing homes initially included in the cohort study, rendering a comparable exposure probability to SARS-CoV-2 (see also Appendix S1 of the manuscript). The anonimity of participants was fully respected. Indeed, it was not possible to test the effect of other co-variables.
Blinding	Because of the nature of this field cohort study, no intervention on the choice of vaccination (non-vaccinated, 1D-vaccinate and 2D-vaccinated) was made for each participant. The sampling system was fully designed to allow anonymity of individual results. The only link between a sample and a test result is the barcode number on the saliva sample tube. The barcodes of the saliva samples of a nursing home were scanned at the time of submission to ensure traceability of the results. On the basis of this operation, it is possible to match anonymous saliva results with a specific nursing home, and thus obtain information on the positivity rate within this establishment. Scanning the barcodes does not permit to identify the person to whom a sample belongs.

Reporting for specific materials, systems and methods

We require information from authors about some types of materials, experimental systems and methods used in many studies. Here, indicate whether each material, system or method listed is relevant to your study. If you are not sure if a list item applies to your research, read the appropriate section before selecting a response.

Materials & experimental systems

n/a	Involved in the study	
☒	☐	Antibodies
☒	☐	Eukaryotic cell lines
☒	☐	Palaeontology and archaeology
☒	☐	Animals and other organisms
☐	☒	Human research participants
☒	☐	Clinical data
☒	☐	Dual use research of concern

Methods

n/a	Involved in the study	
☒	☐	ChIP-seq
☒	☐	Flow cytometry
☒	☐	MRI-based neuroimaging

Human research participants

Policy information about studies involving human research participants

Population characteristics	At the time of this study, there were 572 nursing homes active in the Walloon region of Belgium, 99 of which participated in the cohort study. In these 99 nursing homes, the number of residents was estimated at 7651 individuals. Based on a ratio of 20.5 equivalent full-time workers for 30 residents [Rombeaux JM, 2017], the estimated study population of nursing home workers was around 5228 adults.
Recruitment	The vaccination in the nursing homes was recommended by the Walloon Agency for a Quality Life (AViQ), on a voluntary basis. During the study period, only the BNT162b2 (Pfizer-BioNTech) COVID-19 vaccine was administered to the nursing

home workers [Lamb YN, 2021].

Ethics oversight	The study was approved by the Ethical Committee of the University of Liège (reference number: 2021-101).

Note that full information on the approval of the study protocol must also be provided in the manuscript.
